# Critical uterine bleeding after miscarriage in a warfarin-anticoagulated patient with mechanical heart valve: a case report

**DOI:** 10.1186/s13256-026-05991-3

**Published:** 2026-04-02

**Authors:** Melanie Poloczek, Barbara Kipp

**Affiliations:** 1 Department of Obstetrics and Gynecology, Klinikum Dortmund, Beurhausstr. 40, 44137 Dortmund, Germany; 2 Department of Health, Witten/Herdecke University, Witten, Germany

**Keywords:** Mechanical heart valve, Vitamin K antagonists, Miscarriage, Uterine bleeding, Anticoagulation, Uterine artery embolization, Endometrial ablation, Case report

## Abstract

**Background:**

Patients with mechanical heart valves require lifelong oral anticoagulation with vitamin K antagonists such as warfarin to prevent valve-associated thromboembolism. Bleeding complications during this therapy pose significant clinical challenges and necessitate expert management. In particular, heavy uterine bleeding following miscarriage represents a high-risk and emergent situation. The management of uterine bleeding is challenging, due to the need to balance anticoagulation with hemostatic control. This report presents a case of severe and prolonged bleeding caused by retained product of conception in a patient with mechanical heart valves receiving vitamin K antagonist therapy.

**Case presentation:**

We describe the case of a 28-year-old African woman with mechanical double heart valve prosthesis maintained on warfarin therapy who initially underwent vacuum curettage for a missed abortion. Two weeks later, she presented with heavy vaginal bleeding. Despite initial expectant management, recurrent bleeding episodes necessitated multiple interventions including repeat curettage, off-label intrauterine tamponade, blood transfusions, and bridging with unfractionated heparin. Persistent hemorrhage led to attempted endometrial ablation, followed by successful uterine artery embolization, which effectively controlled hemorrhage and preserved the uterus. The patient was closely monitored from both a cardiologic and hemostatic perspective, and anticoagulation therapy was resumed with stable prosthetic valve function observed at discharge.

**Conclusions:**

The management of miscarriage-associated bleeding in patients receiving warfarin anticoagulation for mechanical heart valves is complex and necessitates interdisciplinary collaboration. A stepwise multimodal approach incorporating gynecological, hematological, radiological, and cardiological expertise can achieve hemorrhage control while maintaining an appropriate balance between thrombotic and bleeding risks. Organ-preserving interventions such as uterine artery embolization may offer viable alternatives to hysterectomy in selected cases.

## Background

Early pregnancy loss is common, affecting approximately 15% of all pregnancies [1]. Vaginal bleeding is one of the most frequent symptoms of miscarriage [2, 3]. Women with mechanical heart valve (MHV) prostheses represent a rare but high-risk subgroup among pregnant individuals. These patients face an increased risk of first-trimester miscarriage as well as serious maternal complications [4]. Notably, only 58% of pregnancies in women with MHVs result in live births without major complications [5]. A key contributing factor is the lifelong requirement for therapeutic anticoagulation with vitamin K antagonists (VKAs) to prevent thromboembolic events [5].

Uterine bleeding in patients with MHVs presents a unique clinical challenge, as the need for anticoagulation to prevent cardiac complications conflicts with the necessity of hemostatic management. While previous studies have addressed peripartum hemorrhage or uterine bleeding in non-pregnant women with MHV [6, 7], there are limited data on miscarriage-related bleeding in this population. We present the case of a patient with mechanical heart valve prostheses who developed persistent heavy vaginal bleeding due to retained products of conception.

## Case presentation

A 28-year-old G3P1 African woman presented with vaginal bleeding two weeks following an uncomplicated vacuum curettage for a missed abortion at 10 weeks gestation. She was receiving warfarin anticoagulation (1–1.5 mg/day, adjusted according to INR) due to a mechanical double valve replacement performed 9 years earlier. Her obstetric history included a spontaneous delivery 12 years ago and a prior abortion managed by curettage 8 years ago.

At initial presentation, the patient was hemodynamically stable with a hemoglobin level of 10.4 g/dl and β-HCG level of 372 U/l. Vaginal examination revealed a clot, but no active uterine bleeding. Transvaginal ultrasound revealed a 13-mm-thickened endometrium, raising suspicion of retained products of conception. The patient was initially managed expectantly.

**Fig. 1:**
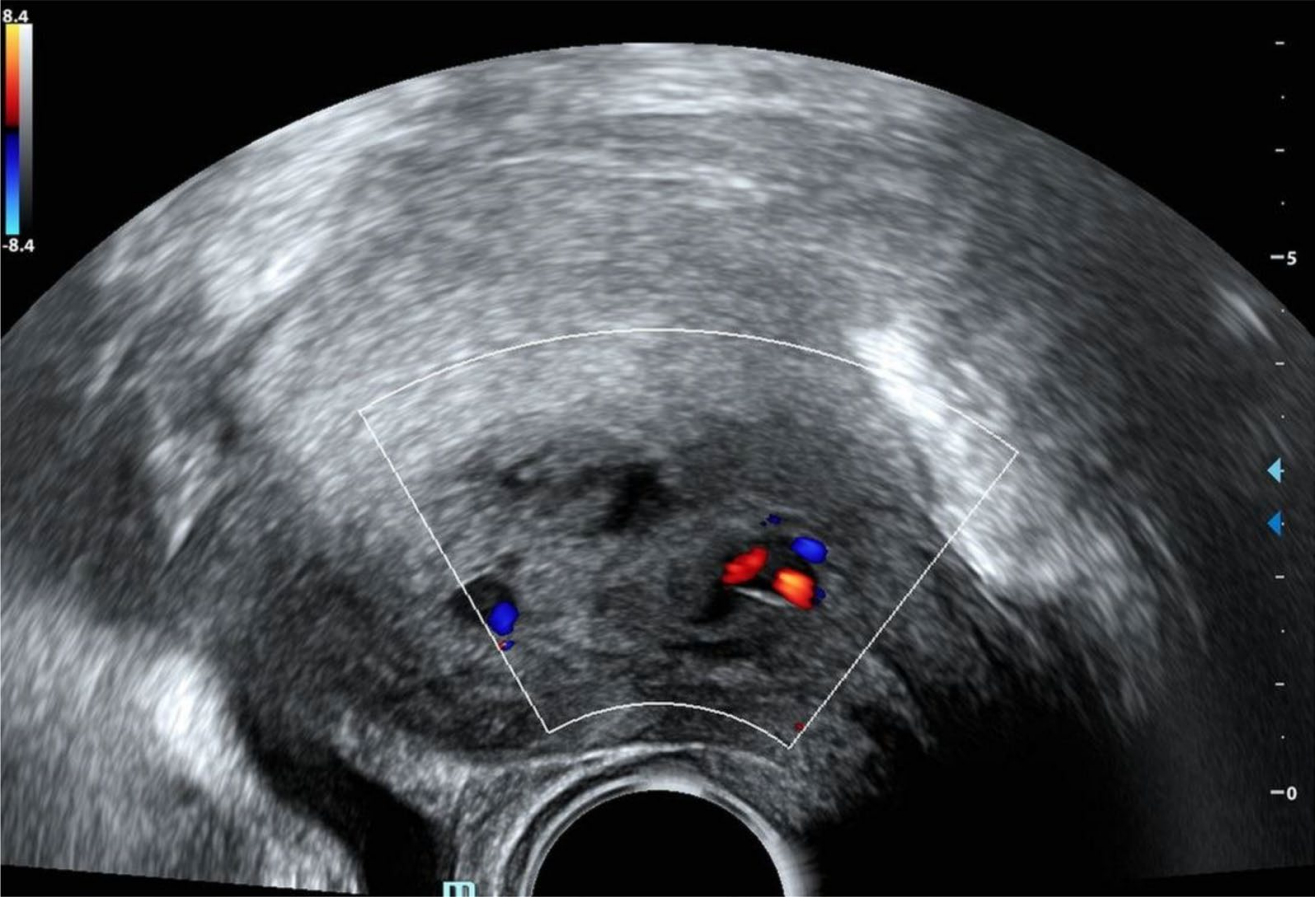
Transvaginal ultrasound at admission demonstrating an intrauterine clot with areas of perfusion

Two days later, she was readmitted with heavy bleeding and a syncopal episode at home. Laboratory tests revealed a drop in hemoglobin to 8.4 g/dl, β-HCG decreased to 196 U/l, INR was elevated at 3.48, and thrombocytopenia was noted with a platelet count of 92,000/µl. Ultrasound demonstrated a 5 × 4 cm intrauterine clot with focal perfusion suggestive of active bleeding (Fig. 1). The patient remained hemodynamically stable (BP 120/80 mmHg, HR 95/min, pO2 100%).

Bridging therapy was started with unfractionated heparin (target aPTT 60–70s). Due to ongoing bleeding, uterine curettage was performed one day after admission. Preoperatively, coagulation was stabilized with 1000 IU of Prothrombin Complex Concentrate (PCC), 2 mg vitamin K, and transfusion of 2 units of red blood cells concentrate (RBC) and 1 unit of platelet concentrate (PC). During the procedure, a chitosan-impregnated tamponade (CT, “Celox™ PPH”) was inserted off-label, leading to effective bleeding control. Histology analysis of the curettage specimen confirmed retained products of conception.

The tamponade was removed one day postoperatively, with minimal bleeding thereafter. A heparin-induced thrombocytopenia (HIT) rapid test was negative despite persistent thrombocytopenia, attributed to consumption. Between days 3 and 6, moderate bleeding continued with hemoglobin dropping to 6.5 g/dl, necessitating further transfusions and continued heparin anticoagulation.

On day 7, the patient experienced recurrent heavy uterine bleeding with clot passage and sonographic evidence of a large intrauterine hematoma. Management included transfusions of RBC and PC, administration of tranexamic acid (TXA), followed by repeat curettage and reinsertion of chitosan tamponade under general anesthesia. Postoperatively, she was transferred to the intensive care unit with a hemoglobin level of 7.4 g/dl.

Due to ongoing bleeding and the patient’s lack of desire for future fertility, definitive therapy was pursued. On day 9, high-frequency endometrial ablation using the NovaSure system was attempted but not completed, as the pre-procedural perforation test failed due to cervical insufficiency. CT was reinserted, and as a last resort prior to hysterectomy, uterine artery embolization (UAE) was performed successfully by interventional radiology.

**Fig. 2:**
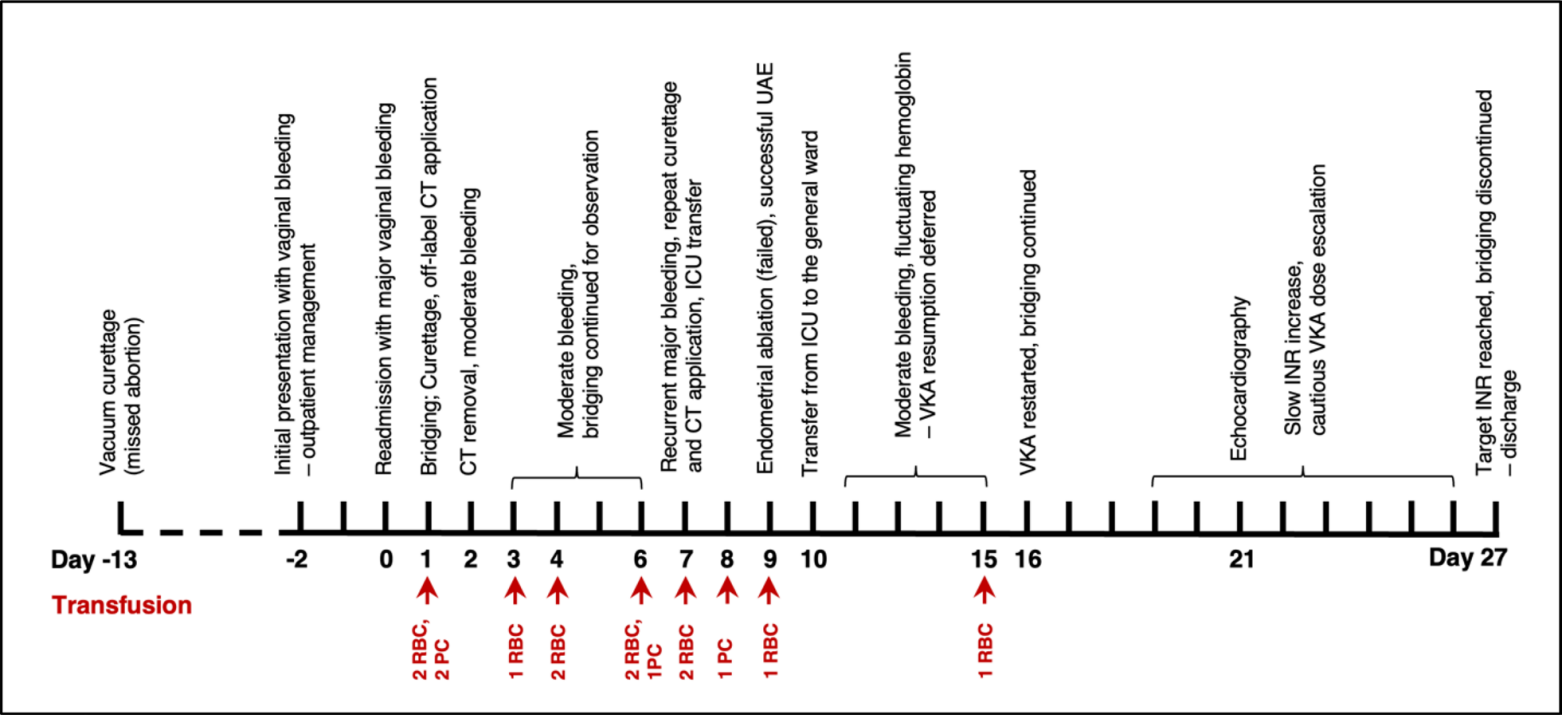
Case report timeline. According to CARE guidelines

Following embolization, bleeding was significantly reduced and stabilized. During hospitalization, the patient received a total of 11 RBC and 4 PC transfusions. Warfarin therapy was resumed from day 17 with temporary heparin bridging. Dose escalation was performed over several days until a target dose of 1 mg/day was reached. Prior to discharge, transthoracic echocardiography revealed no new cardiac abnormalities. The patient was discharged on day 27 after achieving a target INR of 3.0 and discontinuation of heparin. Transvaginal ultrasound at discharge showed a residual 25 mm clot without perfusion. Follow-up was arranged with the outpatient gynecology. The case timeline is shown in Fig. 2.

## Discussion

Pregnant women with MHVs represent a rare but high-risk population. The prevalence of pregnant women with MHVs is difficult to quantify. In a retrospective study by Ng et al. 39,871,862 birth hospitalizations were analyzed, identifying 4,152 women with mechanical heart valves—corresponding to a prevalence of 0.01% [8]. However, valvular heart disease accounts for 30–50% of cardiac conditions during pregnancy with acquired valvular defects responsible for approximately 15% of associated cardiac complications [9].

Prosthetic heart valves are associated with an elevated thromboembolic risk, which is further amplified during pregnancy. Patients with MHV therefore require lifelong therapeutic anticoagulation with vitamin K antagonists [10]. Without anticoagulation, the risk of major embolism is approximately 4 per 100 patient-years but can be reduced to 1 per 100 patient-years with adequate VKA therapy [11].

For pregnant women with MHVs, warfarin remains the only anticoagulant approved for use, as direct oral anticoagulants (DOACs) are contraindicated. VKAs are considered the anticoagulant of choice during pregnancy due to their proven efficacy and the lowest risk of adverse maternal outcomes. Therapeutic monitoring is performed via INR measurements, with an INR target range around 2.5 [10, 12].

However, it is important to note that VKAs are associated with an increased risk of adverse fetal outcomes, including miscarriage and fetal malformations—particularly during the first trimester. These effects, collectively referred to as “fetal warfarin syndrome,” have primarily been observed with daily warfarin doses exceeding 5 mg. Below this threshold, no significant increase in adverse fetal events has been demonstrated [13]. In fact, fetal outcomes were more favorable in patients who received lower warfarin doses targeting a reduced INR range (1.5–2.5) [14]. In contrast, guidelines such as those from the ACC/AHA clearly recommend a high target INR of 2.5 [10].

While anticoagulation remains essential from a cardiac standpoint, this benefit is counterbalanced by an increased risk of abnormal uterine bleeding in women receiving therapeutic anticoagulation [15]. Major bleeding is generally defined as symptomatic bleeding and/or a hemoglobin drop of ≥ 2 g/dl, or the need for transfusion of at least two units of red blood cells [16]. In patients with MHVs, the incidence of major bleeding ranges from 0.34 to 2.91 per 100 patient-years, depending on additional risk factors such as age, INR level, or history of previous bleeding [11].

**Fig. 3:**
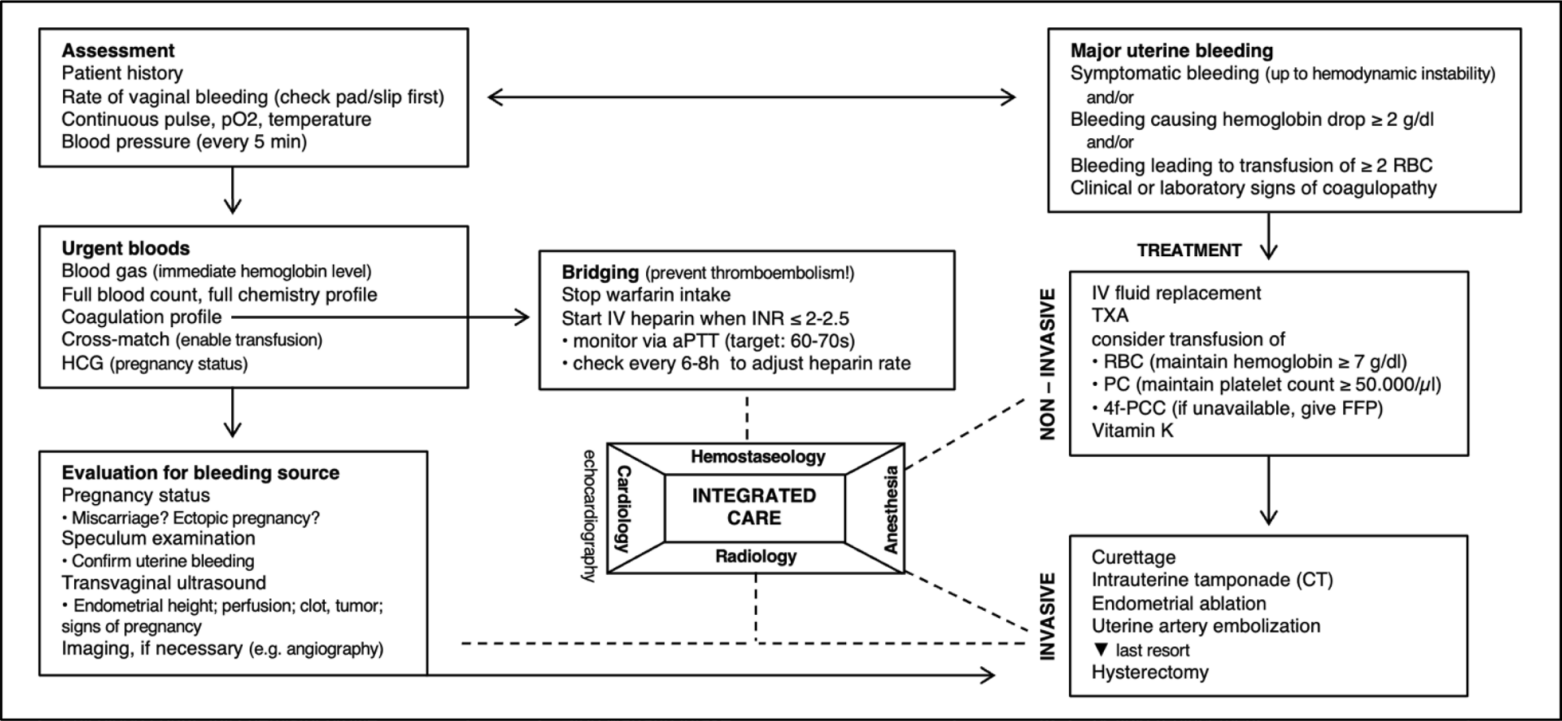
Algorithm for management of miscarriage-related bleeding in patients with MHV under VKA therapy (diagram created by the authors)

Hemorrhage control under VKA therapy thus represents a clinical challenge. Treatment options range from pharmacological management to invasive gynecological procedures. In patients with potential future pregnancy, an escalation scheme is recommended, with hysterectomy as a last resort due to its high bleeding risk and implications for future fertility and operative complications (Fig. 3).

### Non-invasive treatment

#### Bridging

The safe duration for warfarin interruption in patients with MHVs remains unclear due to a lack of large prospective studies. Most available data focus on anticoagulated patients with intracranial hemorrhage, where temporary interruption of anticoagulation for up to two weeks has in some cases been considered acceptable with respect to both thromboembolic and bleeding risks [11].

Regarding the use of bridging after VKA discontinuation, many studies exclude patients with active bleeding. Similarly, the ACC/AHA guidelines provide recommendations for perioperative bridging in patients with MHVs and additional thromboembolic risk factors—which, in our view, must include pregnancy—but these apply to planned interruptions prior to elective interventions [10]. In such cases, intravenous unfractionated heparin (UFH) is typically initiated once the INR falls below the therapeutic range of 2.0–2.5. Therapy should then be monitored using aPTT, targeting values greater than twice the control (~ 60–70 s) [10].

A supratherapeutic INR reflects an imbalanced coagulation state and may either cause or exacerbate major bleeding. Consequently, neither VKA nor UFH should be administered while the INR exceeds the therapeutic range [10]. In addition to discontinuing warfarin and considering bridging therapy once the INR falls below target, further pharmacological and interventional strategies may be warranted.

#### Tranexamic acid (TXA)

Despite the absence of large studies on antifibrinolytic use during anticoagulation, TXA is considered inevitable in major bleeding. Although TXA has been suggested to increase the risk of thromboembolism, multiple studies—including those involving patients with postpartum hemorrhage—have not confirmed this association [17].

#### Transfusion of blood products

RBC and PC transfusions should be administered during active bleeding, aiming to maintain hemoglobin > 7.0 g/dl and platelet count > 50,000/µl [18]. Accordingly, management must take place in settings with immediate access to a well-stocked blood bank.

Coagulation factor concentrates should be considered, preferably 4-factor prothrombin complex concentrate (4f-PCC). If unavailable, fresh frozen plasma (FFP) is an alternative [18]. FFP contains all coagulation factors but must be ABO-compatible and thawed prior to administration [11]. PCC is preferred due to higher concentrations of vitamin K-dependent factors II, VII, IX, X, and proteins C and S, enabling lower infusion volumes and fast application [11, 18].

#### Vitamin K

In cases of ongoing bleeding after PCC administration, vitamin K may be considered for VKA reversal [10]. Disadvantages include delayed biochemical response (4–6 h) and the need for slow intravenous administration [11, 18].

### Invasive treatment

#### Curettage

In cases of early pregnancy loss accompanied by heavy bleeding, curettage is the procedure of choice [19]. Surgical interventions carry an increased risk of bleeding when performed under bridging anticoagulation, although bridging aims to reduce the risk of thromboembolic events [10]. At the same time, curettage under general anesthesia offers the opportunity for pain-free insertion of intrauterine devices if initial curettage is insufficient.

#### Chitosan-impregnated tamponade (CT, “Celox™ PPH”)

Off-label intrauterine application of chitosan-impregnated gauze may be used, providing mechanical tamponade and promoting local hemostasis via erythrocyte membrane interaction. Therefore, CT is well established in postpartum hemorrhage management [20]. Animal studies have already shown its effectiveness in managing arterial bleeding during warfarin therapy [21].

#### Endometrial ablation

Rollerball endometrial ablation has been reported as curative in severe uterine bleeding, including cases under warfarin therapy [22]. Other techniques such as NovaSure radiofrequency ablation and thermal balloon ablation are also described as curative options in acute uterine bleeding disorders [23, 24]. If available, hysteroscopic rollerball ablation may be preferred, since NovaSure ablation proved ineffective in our case due to a gaping cervix, as discussed above.

#### Uterine artery embolization (UAE)

UAE is a safe and effective method to control bleeding in abnormal uterine bleeding of various etiologies [25], including anticoagulation-associated bleeding [26]. UAE has been described as an effective treatment for persistent bleeding after abortion curettage [27]. UAE requires specialized interventional radiology expertise and is thus not universally accessible.

#### Hysterectomy

Hysterectomy is the most invasive procedure and requires the above-mentioned perioperative interruption of anticoagulation. It carries the highest risk of bleeding as well as of venous thromboembolism and should therefore be considered a last resort [28, 29]. Future fertility desires must also be taken into account.

Despite the strategies outlined above, data on the optimal management of bleeding complications in this unique population remain scarce. Most recommendations are based on case reports or extrapolated from non-obstetric populations. Clear data on safe INR thresholds, timing and protocols for bridging therapy, and the efficacy of various hemostatic interventions are lacking. This underscores the urgent need for prospective studies and the development of standardized treatment algorithms tailored to this high-risk population.

## Conclusion

This case highlights the significant challenges associated with managing miscarriage-related bleeding in patients with mechanical heart valves (MHV) undergoing vitamin K antagonist (VKA) therapy. Management requires a careful balance between maintaining anticoagulation to prevent valve thrombosis and achieving timely hemorrhage control. A multimodal, individualized strategy—including coagulation reversal, transfusions, intrauterine tamponade, and uterine artery embolization—is essential to avoid hysterectomy. Close interdisciplinary collaboration is crucial for optimal outcomes.

## Data Availability

Not applicable.

## Abbreviations

4f-PCC: 4-Factor prothrombin complex concentrate
aPTT: Activated partial thromboplastin time
CT: Chitosan-impregnated tamponade
FFP: Fresh frozen plasma
INR: International normalized ratio
MHV: Mechanical heart valve
PC: Platelet concentrate
RBC: Red blood cells concentrate
TXA: Tranexamic acid
UAE: Uterine artery embolization
VKA: Vitamin K antagonists
